# Controllable Introduction of Surface Defects on CH_3_NH_3_PbI_3_ Perovskite

**DOI:** 10.3390/nano12061002

**Published:** 2022-03-18

**Authors:** Sushu Wan, Yajie Zhu, Daocheng Hong, Yuxi Tian

**Affiliations:** 1 Key Laboratory of Mesoscopic Chemistry of MOE, Jiangsu Key Laboratory of Vehicle Emissions Control, School of Chemistry and Chemical Engineering, Nanjing University, Nanjing 210023, China; wansushu@outlook.com (S.W.); yajie.zhu@smail.nju.edu.cn (Y.Z.); hdaocheng@gmail.com (D.H.); 2 Key Laboratory for Advanced Technology in Environmental Protection of Jiangsu Province, Yancheng Institute of Technology, Yancheng 224051, China

**Keywords:** lead halide perovskite, polycrystalline thin films, defect modification, defect concentration, optical microscopy

## Abstract

One of the unique characteristics of semiconductors is the strong dependence of their properties on crystal defects and doping. However, due to the species diversity and low density, it is very difficult to control the type and concentration of the defects. In perovskite materials, crystal defects are randomly formed during the fast crystallization process, causing large heterogeneity of the samples. Here, in this work, we report a controllable method to introduce surface defects on CH_3_NH_3_PbI_3_ perovskite materials via the interaction with 1,4-benzoquinone (BQ) molecules on the gas and solid interface. After the adsorption of BQ molecules on the perovskite surface, surface defects can be generated by photoinduced chemical reactions. The concentration of the defects can thus be controlled by precisely regulating the laser irradiation time. The concentration of the defects can be characterized by a gradually decreased PL intensity and lifetime and was found to influence the atmospheric response and the subsequent acetone-induced degradation of the materials. These results demonstrate that crystal defects in perovskite materials can be controllably introduced, which provides a possible way to fully understand the correlation between the nature and chemical structure of these defects.

## 1. Introduction

Organometal halide perovskites have demonstrated prompt progress in optoelectronic devices [1], e.g., solar cells [2,3,4], light-emitting diodes [5] and lasers [6], thanks to their low-cost preparation method, suitable and tunable bandgap, high absorption coefficient and long charge diffusion length [7,8,9,10,11]. Accompanied by the remarkable increase in the power conversion efficiency (PCE) of perovskite solar cells (PSCs) [12], one main concern of its practical application is the instability of the devices outside the glove box [13]. To improve the device efficiency and lifetime, many strategies, including fabrication optimization [14,15], composition engineering [16,17] and surface passivation [18,19], have been taken, and the modification of defect properties plays an important role during those processes.

Although halide perovskite is reported to demonstrate a high defect tolerance [20,21], it is unavoidable to form deep defects during the solution processing, thus hindering the devices’ PCE approaching the theoretical limit [22]. Aristidou et al. reported that iodine vacancies can mediate the formation of superoxide and induce the degradation of the materials [23]. Azpiroz et al. reported that defects can facilitate ion migration, causing hysteresis effects in PSCs [24]. Grain boundaries are especially vulnerable to forming undercoordinated lead ions and halide vacancies, thus triggering the materials’ degradation [25]. To passivate surface traps, various reagents [15,18,19,26], including ammonium or halide ions and other electron-donating ligands, were developed and proven to efficiently increase the PL lifetime of the materials. Those researches indicate that charge carrier dynamics, the photoluminescence quantum yield (PLQY) and the material stability are strongly related to defects on the surface and inside the perovskite bulks.

Though theoretical work has provided many insights on intrinsic defects and extrinsic dopants and their influence on perovskites’ optoelectronic properties [27,28,29], experimental methods have a limited capability to characterize the chemical structure of defect species due to the great variation of defect properties. Therefore, the correlation between specific defects and their influence on devices is still under debate; much progress would be made if one could control the type and concentration of defects in perovskite materials. In this work, we used inert gas nitrogen (N_2_) to bring moderate oxidant BQ onto the surface of MAPbI_3_ and precisely controlled the oxidation reaction under laser irradiation. We used the PL intensity after treatment as an indicator of the concentration of defects, which can be precisely controlled by the laser irradiation time. Perovskites’ thin films of different defects concentration manifested different PL responses to oxygen (O_2_) and acetone atmosphere. These results prove that the introduced surface vacancies are potential catalysts for redox reactions and indicate that the controllable introduction of defects can help us to gain more insights on defect properties and their impacts on perovskite optoelectronic devices.

## 2. Materials and Methods

### 2.1. Sample Preparation

We used a one-step spin-coated deposition method to prepare MAPbI_3_ thin films [30]. The precursor solution was prepared by adding equimolar methylammonium iodide (CH_3_NH_3_I, MAI, Xi’an p-OLED, Xi’an, China) and lead iodide (PbI_2_, Sigma–Aldrich (Shanghai, China) Co. Ltd., Shanghai, China) mixtures in γ-butyrolactone (Aladdin, Shanghai, China) and stirring the mixture at 80 °C overnight. MAPbI_3_ films were fabricated by spin-coating the 40 μL solution on a cleaned glass substrate (22 × 22 mm) at 3000 rpm and then annealing at 80 °C for 10 min. The morphology of our samples are polycrystalline thin films of submicron-sized crystals, as shown in Figure S1a. All preparation procedures were carried out in the air. 1,4-benzoquinone was purchased from TCI Shanghai Co. Ltd. (Shanghai, China). During the BQ vapor treatment, to minimize the influence of spatial and sample heterogeneity of trap densities, we always compared adjacent regions on the same sample.

### 2.2. Photoluminescence Measurements

All PL kinetics and spectra were measured with a home-built wide-field microscope based on an inverted microscope (Olympus IX73, Olympus (China) Co., Ltd., Beijing, China). The PL signal was collected by a dry objective lens (Olympus LUCPlanFI 40×, NA = 0.6) and recorded by an EM-CCD camera (iXon Ultra 888, Andor Tech., Belfase, UK) after passing through a 550 nm long-pass filter (ET550lp, Chroma Tech. Crop., Bellows Falls, VT, USA). MAPbI_3_ samples were excited with a 532 nm diode laser (MGL-III-532, CNI Optoelectronics Tech., Changchun, China). The PL spectrum was measured by adding a grating in front of the detector. During the PL measurement, the atmosphere was switched between N_2_, O_2_ and BQ/N_2_, according to the experimental requirements, and the gas flow rate was controlled by a gas flowmeter.

### 2.3. Lifetime Measurements

We used a pulsed supercontinuum white laser (WL-SC400, Fianium, Southampton, UK) with AOTFs to obtain a monochromatic light of the selected wavelength. A fast avalanche photodiode (APD, MicroPhoton Devices, Bolzano, Italy) coupled with a time-correlated single photon counting module (PicoHarp 300, PicoQuant, Berlin, Germany) was used for the time-resolved PL decay measurements. The PL decay kinetics were fitted by a bi-exponential decay model and the intensity-averaged lifetimes were extracted from the fitting results.

## 3. Results and Discussion

### 3.1. Solution Treatment vs. Vapor Treatment

In our previous work [31], we proved that the treatment of BQ molecules on the perovskite surface can efficiently introduce MA-I double vacancies via an oxidation reaction between BQ and MAI, causing strong PL quenching. However, taking the PL quenching efficiency after the treatment as an index of introduced defect density, it appears the BQ solution treatment is highly variable, especially at a low BQ concentration (Figure S2 in the Supplementary Materials). This is because although solution treatment can degrade perovskite solids easily and effectively when the concentration of BQ is high, there is a large heterogeneity in the amount of BQ at different positions of the film. To better control the amount of BQ and treat the surface more uniformly, in this work, we developed a vapor treatment procedure by using N_2_ as the carrier gas to bring BQ molecules onto the perovskite surface.

**Figure 1:**
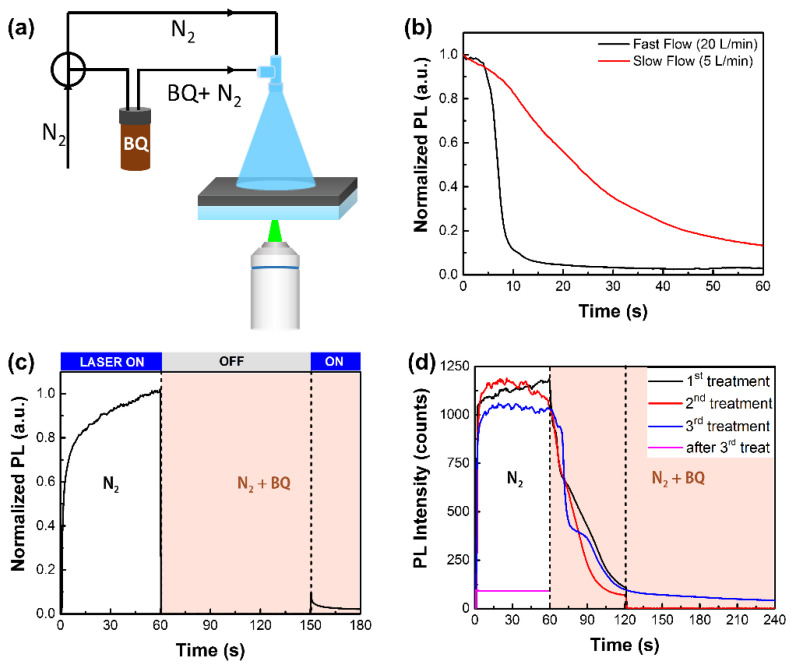
(**a**) Experimental setup of BQ vapor treatment method. By switching the T-valve, we can change the purge atmosphere between pure N_2_ and BQ/N_2_; (**b**) PL-quenching kinetics during BQ vapor treatment of different gas flow velocities demonstrate that PL quenching reaches saturation more quickly with faster flow velocity; (**c**) PL-quenching kinetics show a strong quenching effect of pure BQ adsorption without light irradiation; (**d**) repeated BQ treatment on the same region for different irradiation time. The sample was illuminated for 60 s in the first and second treatment and 180 s in the third one in BQ/N_2_. Only with longer irradiation in the third treatment could PL not recover back to the original level. All above treatment was under the irradiation of 0.2 W/cm^2^ and 532 nm, and the gas flow rate is 5 L per min, besides in Panel b.

The vapor treatment procedure is schematically shown in Figure 1a. Dry N_2_ goes through a gas-washing bottle containing BQ powders and brings volatile BQ molecules to the perovskite thin film. Taking the PL level in pure N_2_ as a reference, we see significant PL quenching once BQ is successfully introduced to the surface, suggesting that the interaction on the gas and solid interface is very effective. The different PL behavior in the N_2_ and BQ vapor also indicates that the rapid PL quenching is due to the introduction of BQ vapor instead of a purely photo-induced PL decay, as reported by others [32]. Thanks to the convenience of the vapor treatment, we can monitor the PL response in situ and in real time. According to the PL kinetics during vapor treatment, the time consumed to reach PL quenching saturation is strongly related to the gas flow speed (see Figure 1b) rather than the excitation intensity (Figure S3), indicating that the fast PL quenching effect is mainly related to the amount of BQ on the surface. To further investigate the effect of light irradiation on the quenching effect of BQ, the illumination was turned off before BQ was purged into the container. As shown in Figure 1c, the PL level has already significantly decreased since we turned the light on, which demonstrates that a strong quenching effect can occur without light illumination. However, when we periodically switched the atmosphere between N_2_ and BQ (Figure S4), it appears that the fast PL-quenching effect is reversible, unlike the effect of the BQ solution treatment.

To determine how to induce an irreversible effect similar to that with the solution treatment, we carried out BQ vapor treatment cycles (Figure 1d) on the same area with different illumination times. In the first two turns of treatment, once the fast decay stage finished (in 60 s), we kept the sample inside the BQ/N_2_ atmosphere for two more min without light irradiation. After that, we used the N_2_ purge for 5 min to remove the intact BQ, then we witnessed a full recovery of the PL level, as expected. However, if we kept the light irradiation for two more min (in the third treatment), N_2_ purge would fail to recover the PL intensity. In summary, the adsorption of BQ molecules on the MAPbI_3_ thin film can quickly and reversibly quench the PL, but only after enough time of a reaction under the light irradiation would the effect become irreversible, showing that the defect introduction process is a photoinduced reaction.

Here, we can attribute the reversible quenching effect to the electron transfer from the conduction band of MAPbI_3_ to BQ. It is reported that charge carriers in MAPbI_3_ can facilitate superoxide formation [33] and BQ is an efficient electron acceptor [34] and superoxide scavenger [35]; thus, electron transfer from MAPbI_3_ to BQ is possible. Charge transfer between perovskites and BQ would quench the PL of the perovskites; however, once BQ leaves the perovskite surface, the PL level can recover back. Under longer illumination, light-induced charge carriers in perovskites can facilitate the reaction between BQ and MAI, as BQ has been reported to be able to oxidize MAI into volatile methylamine and iodine [36]. Thus, with longer irradiation, PL quenching is irreversible because MA–I double vacancy defects have formed on the surface of MAPbI_3_, as discussed elsewhere [31].

### 3.2. Controllable Surface Defects Introduction

**Figure 2:**
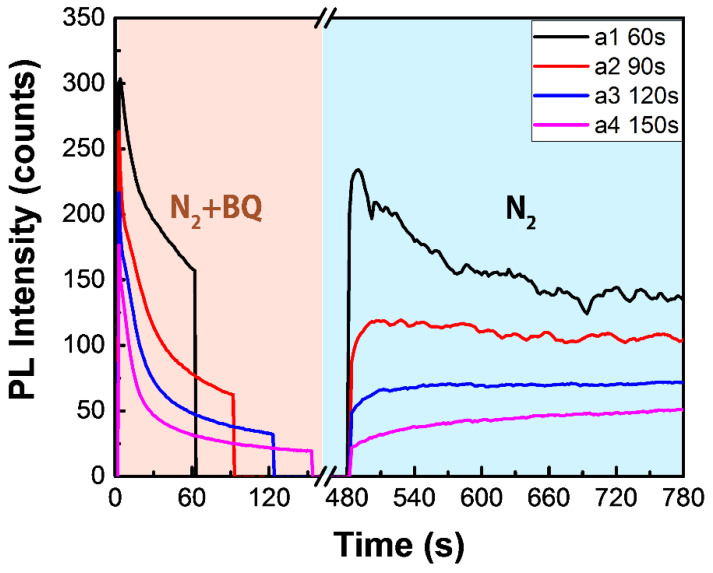
PL kinetics show BQ vapor treatment results with different irradiation durations (at 532 nm) on four areas in the same sample. After BQ vapor treatment, the sample chamber was purged with dry N_2_ for 5 min to remove the residual BQ.

Now, we have a basic understanding of the interaction between BQ molecules and perovskite thin films on the gas and solid interface. By regulating the irradiation duration, we can realize delicate control over the surface defect concentrations. First, we slowed down the gas flow velocity of BQ/N_2_ to 5 L per min with an air flowmeter. After that, we can accelerate the reaction by illumination and control the reaction extent with different irradiation durations. Before checking the treatment results, we shut down the laser and used the N_2_ purge for 5 min to remove intact BQ molecules. By illuminating four different areas with similar initial PL levels for different durations (60 s, 90 s, 120 s and 150 s), we see in Figure 2 that the PL intensity of MAPbI_3_ after N_2_ purging gradually decreased as the irradiation duration increased. We would like to stress that the four areas are adjacent regions on the same film, and we took signals averaged over tens of micrometers in each area to minimize the influence of heterogeneity in the polycrystalline films. As it is feasible to change the irradiation duration, we can easily control the reaction extent to prepare a series of areas of different PL intensities. In addition, by controlling the light irradiation, we can also selectively treat the material on specific locations.

**Figure 3:**
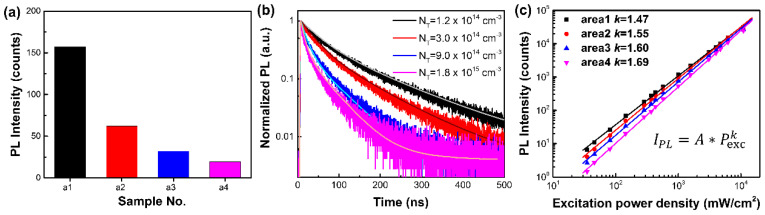
(**a**) PL intensity of four areas after BQ vapor treatment of different irradiation durations; (**b**) time-resolved PL decay kinetics and the fitted results of the four areas. Concentrations of deep traps, *N*_T_, evaluated by the PL decay kinetics, are indicated by the legend. The excitation fluence is 2.5 μJ/cm^2^ (*n*_0_ ~ 3.3 × 10^16^ cm^−3^) at 532 nm, 1 MHz; (**c**) PL intensity (*I_PL_*) as a function of excitation intensity (*P_exc_*). The dots are experimental results, and the solid lines are fitted results using the power law relationship $I_{PL}=A\times P_{exc}^{k}$.

To further verify the effect of the BQ vapor treatment, we conducted a series of spectroscopic measurements on the four treated areas. Both the steady-state PL intensity (Figure 3a) and PL decay lifetime (Figure 3b) prove that we prepared four areas of gradually increased degradation extent with consistent variation trends of PL level and lifetime decreasing (in Figure S5a). The PL lifetime measurement is not influenced by the quenching effect of BQ adsorption, as the intact BQ has been removed with the N_2_ purge. From the PL decay curves, we can estimate the concentrations of the quenching defects to increase by an order of magnitude from the first to the fourth area (details of simulation can be found in the Supplementary Materials). Although MAPbI_3_ thin films experienced significant PL quenching, no spectral shift was observed under different treatment durations (Figure S5b), indicating that the treatment only introduced nonradiative recombination pathways into the crystals rather than destroying them. This can also be verified by no significant variation of the crystal morphology before and after BQ treatment (Figure S1). Furthermore, we measured the PL intensity as a function of the excitation power density, as shown in Figure 3c. The PL intensity (*I_PL_*) generally follows a power law relationship against the excitation power density (*P_exc_*):

*I_PL_* = *A* × *P^k^_exc_* (1)

In the case of MAPbI_3_ measured with a low excitation intensity, the excitation occurs via single photon absorption; thus, the *k* value indicates the dominant radiative recombination dynamics (monomolecular or bimolecular). At room temperature, the dominant carriers in MAPbI_3_ are free charges; thus, the contribution of mono- and bimolecular recombination is related to the trap-filling effect and the density of defects. Wen et al. analyzed in detail that there exists a positive correlation between the density of quenching defects and the *k* value [37]. We can use the *k* value to evaluate the density of defect states after BQ vapor treatment, and the fitting results of the power-dependent PL in Figure 3c show that the *k* value gradually increased in the four areas, indicating a higher defect density induced by the BQ treatment from the first to the fourth area. According to our former results of the BQ solution treatment, the defects introduced by the BQ vapor treatment are probably vacancies of iodine anions (V_I_−) and methylammonium cations (V_MA_+) [31].

According to the above results, we can evaluate the reaction rate of the photo-induced oxidation reaction to get a better control over the defect introduction process. According to the introduced trap density calculated by fitting the PL lifetime (Figure 3b), we evaluated the photo-induced reaction rate on the gas-solid interface to be 3.1 × 10^−8^ mol∙L^−1^∙s^−1^. However, the introduced defects only distributed on the surface region; thus, the actual defect density is even higher than we evaluated here. As reported by Qin et al. [36], the averaged redox reaction rate between BQ and MAI measured by ^1^H-NMR is around 1.9 × 10^−11^ mol∙L^−1^∙s^−1^ in the mixture solution of PbI_2_, MAI and BQ. We noticed that the reaction on the gas-solid interface is more efficient than the spontaneous reaction in the solution, which might be due to the acceleration of reaction by the light irradiation.

### 3.3. Impact of Defect Concentration on the PL Response to O_2_

The PL quenching and enhancement effect of O_2_ on MAPbI_3_ have long been debated. Some observed a photoinduced PL enhancement of MAPbI_3_ and attributed it to adsorption and chemical reactions involving O_2_-related species to passivate defects and suppress nonradiative processes [38,39], while others proposed that superoxide formation would lead to the degradation of MAPbI_3_ to PbI_2_ [23,40]. Comparing regions with and without light irradiation in the BQ vapor treatment, we see a completely reverse atmospheric effect of O_2_, as shown in Figure S6. Furthermore, by preparing areas of different surface defect densities, we simultaneously observed the PL quenching and enhancement effects of O_2_ on the same region and compared their different relationships with the density of MA-I double vacancies. The coexistence of the favorable and detrimental effects of the atmosphere are related to the type, concentration and charge states of crystal defects.

**Figure 4:**
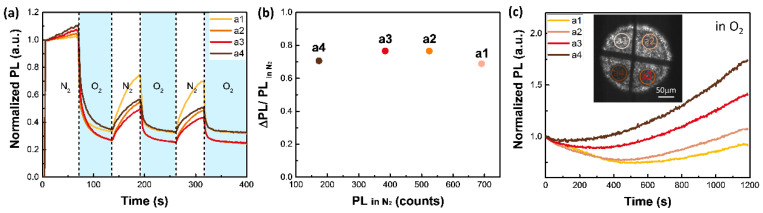
(**a**) PL-quenching effect and (**c**) PL-enhancement effect of O_2_ in the four BQ-treated areas with gradually increasing surface defect densities from the first to the fourth area; (**b**) PL-quenching efficiency of O_2_ in four areas on the same sample extracted from the data in Panel a. The PL level gradually decreased from the first area to the fourth area.

As shown in Figure 4a, all four areas treated with BQ vapor show a fast and strong PL-quenching response to O_2_. Although the four areas with different surface defect densities show gradually decreasing PL levels in dry N_2_, the quenching efficiencies in the O_2_ of those areas show almost no difference, indicating that the PL-quenching effect is related to the intrinsic surface structure as synthesized, rather than the MA-I double vacancies introduced by the BQ treatment. Reversible PL quenching by O_2_ on the time scale of tens of seconds has been observed by others. Some suggested that O_2_ can receive electrons from MAPbI_3_ and quench the excited states [41], while others attributed it to the activation of deep defects inside the bandgap [42]. According to previous results [23,31], electron transfer can be facilitated by iodine vacancies. However, in our case, the introduced iodine vacancy has a trivial influence on the PL-quenching process, indicating that this effect is probably not due to O_2_ as an electron scavenger on iodine vacancies or any other iodine-related species. Therefore, the most suspicious species inducing PL quenching in O_2_ are the Pb-related species, because BQ has little effect on them; thus, their concentrations would not change much after the treatment.

Although MAPbI_3_ shows PL quenching at first, after a longer illumination period, it started showing a PL-enhancement trend in the O_2_ atmosphere, as shown in Figure 4c. The PL intensity variation is determined by the competition between PL quenching and PL enhancement. Interestingly, the PL-enhancement rate increases with increasing defect densities from the first to the fourth region. The consistent trends of the surface defect density and PL enhancement rate prove that PL curing is mediated by vacancies introduced by BQ treatment. However, compared to the fast decay (Figure 1b) resulting from BQ adsorption on the MAPbI_3_ surface, the PL enhancement is much slower on a time scale of tens to hundreds of min, indicating that the interaction between O_2_ and surface defects is more complex than pure adsorption. Previous work from our group [31] and others [39] proposed that the PL curing of O_2_ is mediated by iodine vacancies. It was also theoretically proven that O_2_ molecules energetically prefer to adsorb on iodine vacancies and can receive electrons from the conduction band to form superoxide species [23,43]. Here comes the controversy that some suggested O_2_ adsorption and the formation of superoxide would passivate iodine vacancies and improve the PLQY [39,43], while others proposed that superoxide would cause the degradation of MAPbI_3_ and instability of perovskite devices [23,40]. Our experimental results confirmed that O_2_ would passivate iodine vacancies and improve the PLQY on a time scale of tens to hundreds of min.

### 3.4. Impact of Defect Concentration on the Photocatalysis Reaction

Superoxide is a highly reactive oxidizing species; thus, the light-induced formation of superoxide on perovskite surfaces enables the potential application of this material in photocatalysis. In fact, perovskite-type oxides have long been utilized as photocatalysts for CO_2_ reduction, water splitting and organic pollutant degradation [27,44,45]. Advantages of metal halide perovskites, i.e., efficient charge separation, strong absorption and suitable bandgap, not only make it a good candidate for solar cells but also for photocatalysis [46]. Our previous work demonstrated that BQ-treated MAPbI_3_ can mediate acetone oxidation under light illumination [31]. During the reaction, O_2_ molecules adsorb on iodine vacancies and accept electrons, while acetone adsorb on MA^+^ vacancies and accept holes. Here, we compared the photocatalytic reactivity of MAPbI_3_ micro-areas with different surface defect densities and illustrated the active sites responsible for charge transfer during photocatalysis.

**Figure 5:**
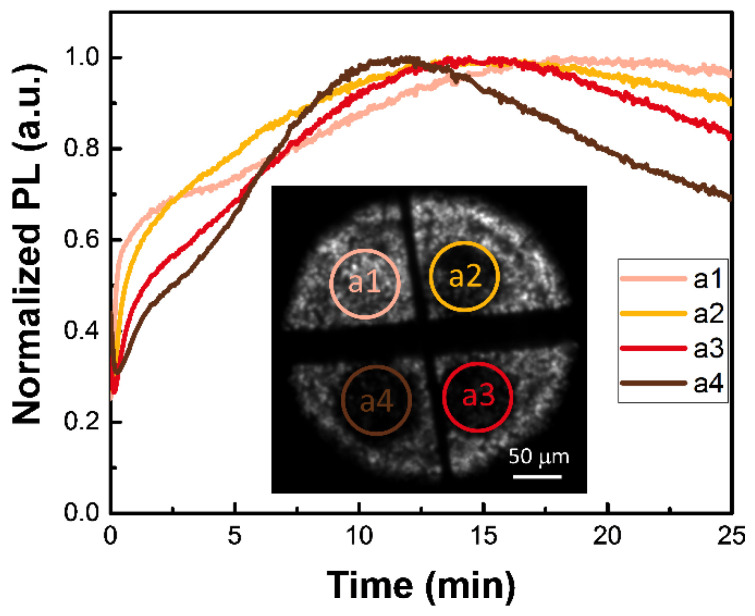
PL responses of the four areas with gradually increasing defect concentration in the mixture of O_2_ and acetone. The inset shows the PL image of four areas (a1–a4) whose PL levels gradually decreased after BQ vapor treatment for different irradiation durations. Only the circled regions were treated under laser irradiation.

Taking advantage of the precise control over surface defects via the BQ vapor treatment, we treated the four areas on the same sample in the BQ vapor for different irradiation durations to check their PL response in the mixture of O_2_ and acetone. At first, both O_2_ and acetone can passivate surface defects, thus inducing PL enhancement. After a while, PL levels started to decrease and the degradation was irreversible, i.e., the perovskite lattice was destroyed. PL images of the treated areas demonstrated that their PL levels gradually decreased, while the degradation rates gradually increased as the defect density increased from the first to the fourth area, as shown in Figure 5. The correlation between the degradation rate and surface vacancy concentration further confirms that MA-I double vacancies are active sites mediating the charge transfer. Combined with the above results that O_2_ can form superoxide on iodine vacancies, more experimental evidence was obtained to prove the Usanovich acid-base passivation mechanism [31] of O_2_ and acetone on the MAPbI_3_ surface.

## 4. Conclusions

In this work, we developed a vapor treatment method to realize precise control of the defect concentration on the MAPbI_3_ surface by a photoinduced chemical reaction with BQ. We found that the adsorption of BQ can cause reversible reduction of the PLQY of MAPbI_3_, but only with laser illumination can MA-I double vacancies be introduced as quenchers. Controllable surface modification is facilitated by controlling the duration of light irradiation. The PL response to oxygen and the photocatalysis reaction of oxygen and acetone were checked in areas with different BQ treatments, demonstrating the dependence of the atmospheric effect on the concentration of surface vacancies. Our experimental results distinguished the origins of PL quenching and the enhancement effect of oxygen on MAPbI_3_ thin films and identified the defect species responsible for the atmospheric effect. This method also illustrates that defects can also be useful in photocatalysis. In the future, we can apply this method to characterize the chemical nature of the surface defects and build up the correlation of defect structures and the photophysical processes mediated by the defects.

## Data Availability

The data is available on reasonable request from the corresponding author.

## Supplementary Materials

The following supporting information can be downloaded at: https://www.mdpi.com/article/10.3390/nano12061002/s1, Figure S1: Morphology of MAPbI_3_ thin films; Figure S2: Effects of BQ solution treatment; Figure S3: Dependence of BQ treatment efficiency on excitation intensity; Figure S4: Reversible PL response in N_2_ and N_2_/BQ mixture; Figure S5: PL spectra before and after the treatment; Figure S6: PL image and response to O_2_ and acetone with and without treatment; Figure S7: Estimation of initial carrier density *n*_0_ and the deep trap density *N*_T_.
