# Supplementary material for: Controllable Introduction of Surface Defects on CH3NH3PbI3 Perovskite
Source: Nanomaterials (Basel). 2022 Mar 18;12(6):1002. doi: 10.3390/nano12061002 (PMC8954356; doi:10.3390/nano12061002)
Supplement: Supplementary file 1 [file nanomaterials-12-01002-s001.zip › nanomaterials-1615297-supplementary.pdf]

nanomaterials

MDPI

Supplementary Materials

Controllable Introduction of Surface Defects on $CH_3NH_3PbI_3$ Perovskite

Sushu Wan [1], Yajie Zhu [1], Daocheng Hong [1,2] and Yuxi Tian [1,*]

[1] Key Laboratory of Mesoscopic Chemistry of MOE, School of Chemistry and Chemical Engineering and Jiangsu Key Laboratory of Vehicle Emissions Control, Nanjing University, Nanjing 210023, China; wansushu@outlook.com (S.W.); yajie.zhu@smail.nju.edu.cn (Y.Z.); hdaocheng@gmail.com (D.H.)
[2] Key Laboratory for Advanced Technology in Environmental Protection of Jiangsu Province, Yancheng Institute of Technology, Yancheng 224051, China
* Correspondence: tyx@nju.edu.cn

1. Morphology of samples with and without BQ treatment

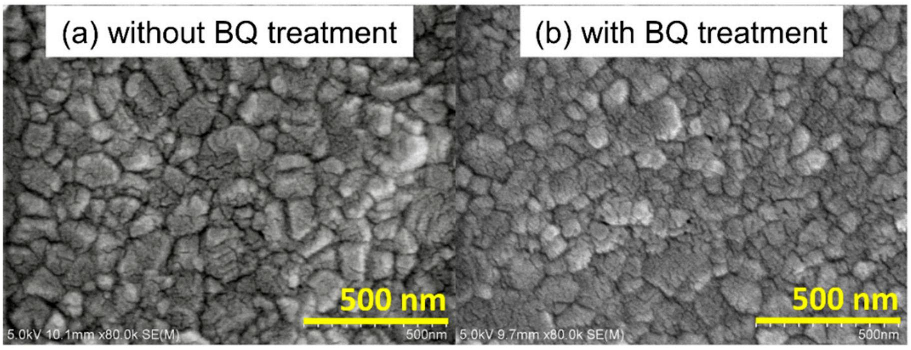

Figure S1. Morphology of $MAPbI_3$ thin films (a) without BQ treatment and (b) with BQ treatment. The thin film consists of submicron-sized polycrystalline. The morphology shows no significant difference with and without BQ treatment indicating that BQ treatment would not destroy the crystal structure.

2. Effects of BQ solution treatment

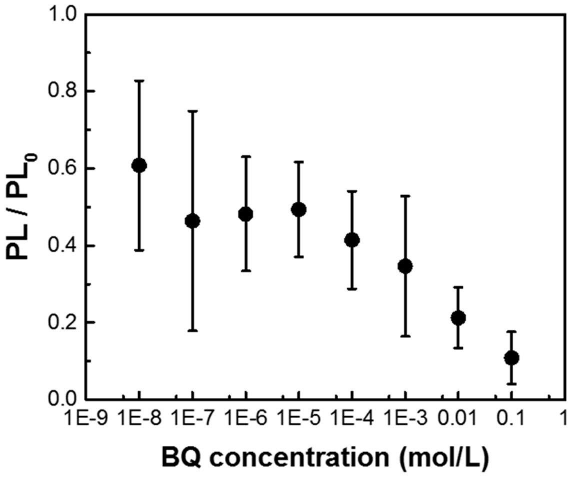

Figure S2. PL quenching efficiency of $MAPbI_3$ thin films when treated with BQ solutions of different concentrations.

3. Dependence of BQ treatment efficiency on excitation intensity

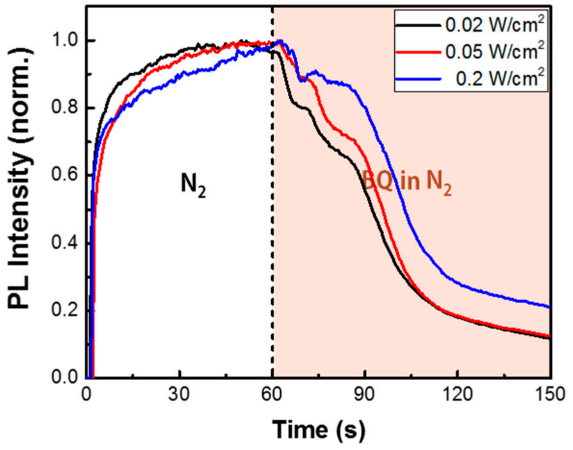

Figure S3. The PL kinetics during BQ vapor treatment under different excitation power. The flow velocity of BQ/N_2 is 5 liters per minute during the measurement.

4. Reversible PL response in N_2 and N_2/BQ mixture

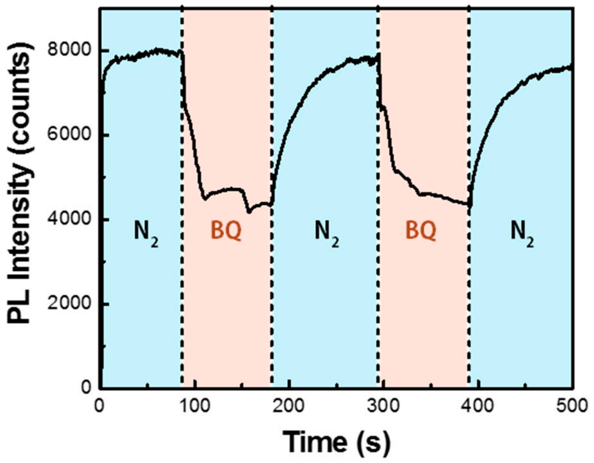

Figure S4. PL kinetics show reversible response when the atmosphere was switched between pure N_2 and BQ/N_2.

5. PL spectra before and after treatment

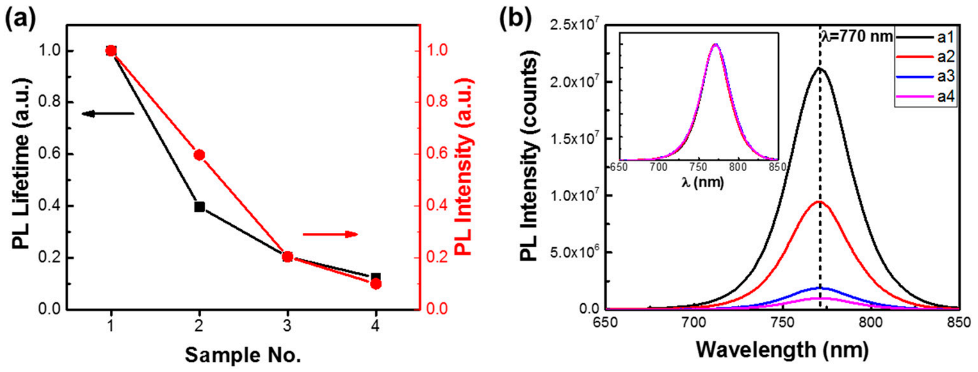

Figure S5. (a) PL lifetime (black square) and PL intensity (red circle) decay trends and (b) PL spectra of four areas in Figure 3a.

6. PL response to O_2 and acetone with and without treatment

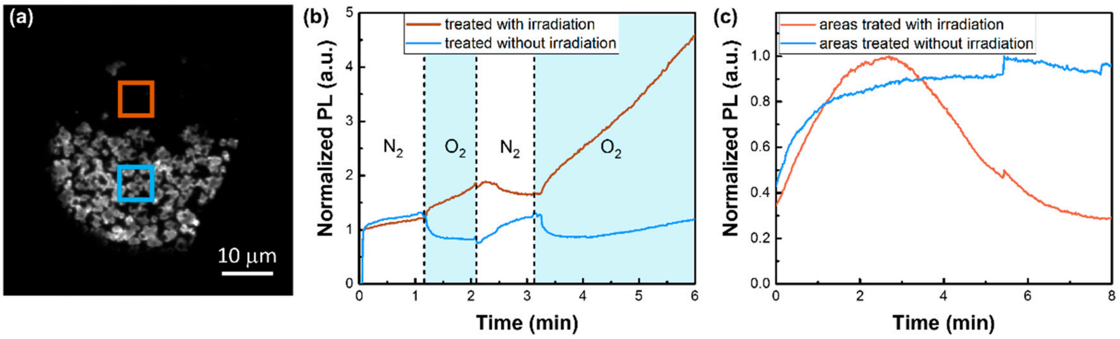

Figure S6. (a) PL image showing the after effect of degradation in the mixture of oxygen and acetone. The upper half was treated with BQ vapor under light irradiation while the lower half without light irradiation; (b) PL response under periodical switching between O_2 and N_2 showing different trends; (c) PL kinetics showing different responses in the mixture of oxygen and acetone. The region treated with light irradiation quickly degraded.

7. Initial carrier density and deep trap concentration

The initial carrier density can be estimated according to the excitation fluence I_0 and absorption coefficient α[1]:

$$n_0 = \frac{I_0(1 - e^{-\alpha d})}{h\nu \cdot d} \tag{1}$$

For polycrystalline thin films containing 2×2×2 μm³ micro-crystals, we can take $1\text{-}e^{-\alpha d}$ approximately as 1. The excitation fluence I_0 of our pulsed laser is 2.5 μJ/cm² and the frequency of 532 nm photon is 5.64×10^{14} s^{-1}. Thus, the initial charge carrier density can be estimated to be 3.3×10^{16} cm^{-3}.

Under such moderate charge carrier density, we can ignore the Auger recombination process and get the differential equation of charge carrier recombination as:

$$\frac{dn}{dt} = -k_1 n - k_2 n^2 - k_{NR} n N_T \tag{2}$$

where k_1 is the monomolecular recombination rate constant, k_2 is the bimolecular recombination rate constant, k_{nr} is the trap-mediated non-radiative recombination rate constant and N_T is the concentration of quenching defects. Taking both mono- and bi-molecular recombination process as radiative, we can get the PL intensity as:

$$\mathrm{PL} = \int (k_1 n + k_2 n^2) dt \tag{3}$$

We did the simulation with parameters same as the reference[2] where $k_1 = 1\times10^6$ s^{-1}, $k_2 = 2.5\times10^{-10}$ cm^3·s^{-1}, $k_{nr} = 2.5\times10^{-10}$ cm^3·s^{-1}. By fitting the PL decay curves with MATLAB, we can get the deep defects densities in the four treated areas.

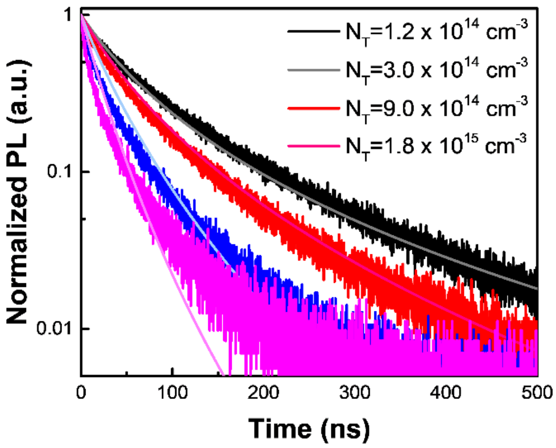

Figure S7. Experimental and fitting results based on the above model of the four treated areas.
